# Children's emotions dataset: Facial images as action units and valence scores

**DOI:** 10.1016/j.dib.2025.112053

**Published:** 2025-09-13

**Authors:** John Barco-Jiménez, Sixto Campaña, Álvaro Cervelión, Harold Cabrera, Carlos Tobar, Roberto Jaramillo, Andrés Diaz, Abel Méndez Porras

**Affiliations:** a Universidad Nacional Abierta y a Distancia, Calle 14 # 28 - 45, Sector Bomboná, Pasto, Nariño, Colombia; b Universidad de Pamplona, Calle 5 # 3 - 93, Pamplona, Norte de Santander, Colombia; c Instituto Tecnológico de Costa Rica, Sur de la Basílica de los Ángeles, C. 15, Provincia de Cartago, Dulce Nombre, Cartago, Costa Rica

**Keywords:** Emotions, Emotional narrative, Classification of emotions, Valences

## Abstract

The paper presents a dataset of emotions from children between 10 and 12 years old. This dataset was obtained from videos that are represented in time series of facial Action Units (AUs), and their corresponding valences were scored by professionals. The AUs are extracted from the videos using the *Deepface* library, and the valence series are obtained from expert observers who rate each video on a range from -1 to 1, covering the spectrum of negative to positive emotions. The dataset was evaluated by a total of 20 professional experts, comprising psychologists and psychology practitioners, with each video receiving an average of 10 reviews. The analysis encompassed a total of 57 videos, representing 22 students, culminating in the acquisition of a comprehensive set comprising 50 temporal series of action units and their associated weighted valence scores. This dataset is useful for training machine learning models in the process of identifying emotions to determine possible patterns of behaviour in classrooms. These patterns may reveal problematic academic attitudes or situations, or, conversely, the early identification of positive emotions that can empower leading students. In addition, it can assist education professionals in undertaking self-evaluations of their formative processes, with a focus on the emotions or attention exhibited by their students within the classroom environment during lessons.

Specifications Table

**Table:** 

Subject	Social Sciences
Specific subject area	Child Psychology and Educational Psychology
Type of data	*Tables in csv format*
Data collection	*The data presented herein were obtained from interviews conducted by psychologists, who employed emotional narratives in their guidance of 22 children. The emotions were evaluated by experts using a continuous valence scale. A unique valence series was obtained for each video by means of weighted averaging of the evaluations performed by the observers. Facial expressions were captured using a camera (1080p, 30FPS), and the AUs were extracted with the DeepFace library.*
Data source location	*Túquerres, Nariño, Colombia, located at the geographical coordinates: 1.0897294° N, 77.6210509° W.*
Data accessibility	Repository name: Children’s Emotional Expression Dataset: Action Units and Valence Time Series available in [1] Data identification number: 10.17632/3fgwr2bwc9.2 Direct URL to data: https://data.mendeley.com/datasets/3fgwr2bwc9/2
Related research article	none

## 1 Value of the Data

• This dataset provides a valuable resource for studying emotional expression in children aged 10 to 12, a demographic that is often underrepresented in research on automatic emotion recognition. This dataset is distinct from other datasets based on adults or acted emotions, in that it captures spontaneous and natural emotional reactions through psychological interviews guided by emotional narratives.
• The data combines continuous valence scores with frame-by-frame extracted facial AUs, enabling the analysis of emotion time series. This approach is particularly beneficial for the training and validation of machine learning models that require the interpretation of emotional dynamics over time.
• The valence ratings were made by a diverse group of experts in psychology, including practicing professionals and advanced students. Furthermore, a consensus error analysis was applied to filter out unreliable samples, thus ensuring the robustness and quality of the emotional labels.
• Researchers in psychology, education, and computer vision can utilise this dataset to develop and validate emotional detection systems, particularly in educational contexts such as classroom monitoring, socio-emotional learning tools, or adaptive educational technologies.
• The methodology used in data collection and validation can be replicated in other cultural contexts or age ranges, facilitating comparative studies and the construction of emotional recognition systems adapted to specific populations.
• This dataset is also useful for the development of human-centered technologies, such as intelligent systems that respond empathetically to the emotional states of children, and for evaluating the effectiveness of educational or clinical interventions with an emotional focus.

## 2 Background

The main motivation for compiling this dataset was the need for specific resources to study the emotions of children in educational contexts, through technological tools and rigorous psychological methodologies [5]. The collection was carried out within the framework of a study that applies the emotional narrative technique, guided by psychologists, to evoke natural emotional responses in children between 10 and 12 years old. This technique, inspired by the SENDv1 methodology of Stanford University [2], allows for the capture of spontaneous emotional expressions through the use of personal experiences. The emotions were recorded on video and evaluated using continuous valence scales by a group of qualified psychologists. At the same time, the "DeepFace" library [3] was used to automatically extract AUs, thereby generating synchronised time series. The resulting dataset has been designed to serve as input in the training and evaluation of machine learning models focused on emotional recognition in children. This data article aims to complement research on the analysis of emotions in school environments, providing a structured and reusable basis for future educational and technological applications.

Facial AUs, defined in the Facial Action Coding System (FACS), represent specific facial muscle movements that can be objectively identified and measured. Each AU is assigned a numerical code corresponding to the contraction or relaxation of one or more facial muscles, such as AU 1 (inner brow raiser) or AU 12 (lip corner puller). In automated facial analysis frameworks such as DeepFace [3], AU activation is expressed both as a binary probability (ranging from 0 to 1, where values closer to 1 indicate a higher likelihood of activation) and as a continuous intensity score that can exceed 1 depending on the strength of the muscle contraction. Negative values are not possible because the metrics represent presence and magnitude, not direction. Occasionally, values above the expected range may appear due to normalization, calibration, or particularly strong facial movements. This quantitative characterization allows researchers and algorithms to differentiate between subtle and pronounced expressions, thereby improving the accuracy of emotion recognition and behavioral analysis.

Table 1 presents the relationship between specific emotions and the corresponding activation patterns of Action Units (AUs), illustrating how different facial muscle movements are associated with distinct emotional expressions.

**Table 1:** AUs to facial expression categories (Extracted from [4]).

Emotion	AU1	AU2	AU4	AU5	AU6	AU9	AU12	AU15	AU17	AU20	AU25	AU26
happy	0.0	0.0	0.0	0.0	0.51	1.0	0	0.0	0.0	0.0	1.0	0.0
surprise	1.0	1.0	0.0	0.66	0.0	0.0	0	0.0	0.0	0.0	0.0	1.0
disgust	0.0	0.0	0.31	0.0	1.8	0.0	0	0.0	0.0	0.13	0.0	0.0
neutral	0.0	0.0	0.0	0.0	0.0	0.0	0	0.0	0.0	0.0	0.0	0.0
fear	1.0	0.57	1.0	0.63	0.0	0.0	0	0.0	0.0	0.0	1.0	0.33
angry	0.0	0.0	1.4	0.8	0.21	0.0	0	0.0	0.67	0.5	0.0	0.0
sadness	0.6	0.0	1.0	0.0	0.5	0.03	0	0.67	0.0	0.0	0.0	0.0

## 3 Data Description

The dataset is organized into two main folders:

1. AUs/ – Action Units Time Series

This folder contains 50 CSV files, each representing a time series of facial Action Units (AUs) extracted from video frames. These AUs act as the input features corresponding to a specific subject and induced emotion.

• **File naming convention**: E##_CT_$_aus.csv. Where: ## is the subject identifier (two-digit student ID), $ is the emotion category = {N: Negative, M: Mixed or Neutral, P: Positive}
• **File content**: Each file is structured as follows:

**Table:** 

**Column**	**Description**

Image	Frame identifier (image number)
AU01_r to AU25_r	Twelve AU values per frame (AU01_r, AU02_r, AU04_r, AU05_r, AU06_r, AU07_r, AU12_r, AU14_r, AU15_r, AU17_r, AU20_r, AU25_r)

• **Number of rows**: Varies per file and corresponds to the number of video frames (time samples) in the series.

2. Valences/ – Valence Time Series

This folder also contains 50 CSV files, each corresponding to the valence values associated with the same video sessions represented in the AUs/ folder.

• **File naming convention**: E##_CT_$_valences.csv. This naming pattern matches the corresponding AU file, enabling easy file pairing.
• **File content**: Each file includes a single column with the valence values over time.

**Table:** 

**Column Name**	**Description**

E##_CT_$	Valence value per time sample

• **Number of rows**: Matches exactly the corresponding AU file to ensure one-to-one alignment between facial expression features and valence data. All paired files are synchronized.

**Fig. 1:**
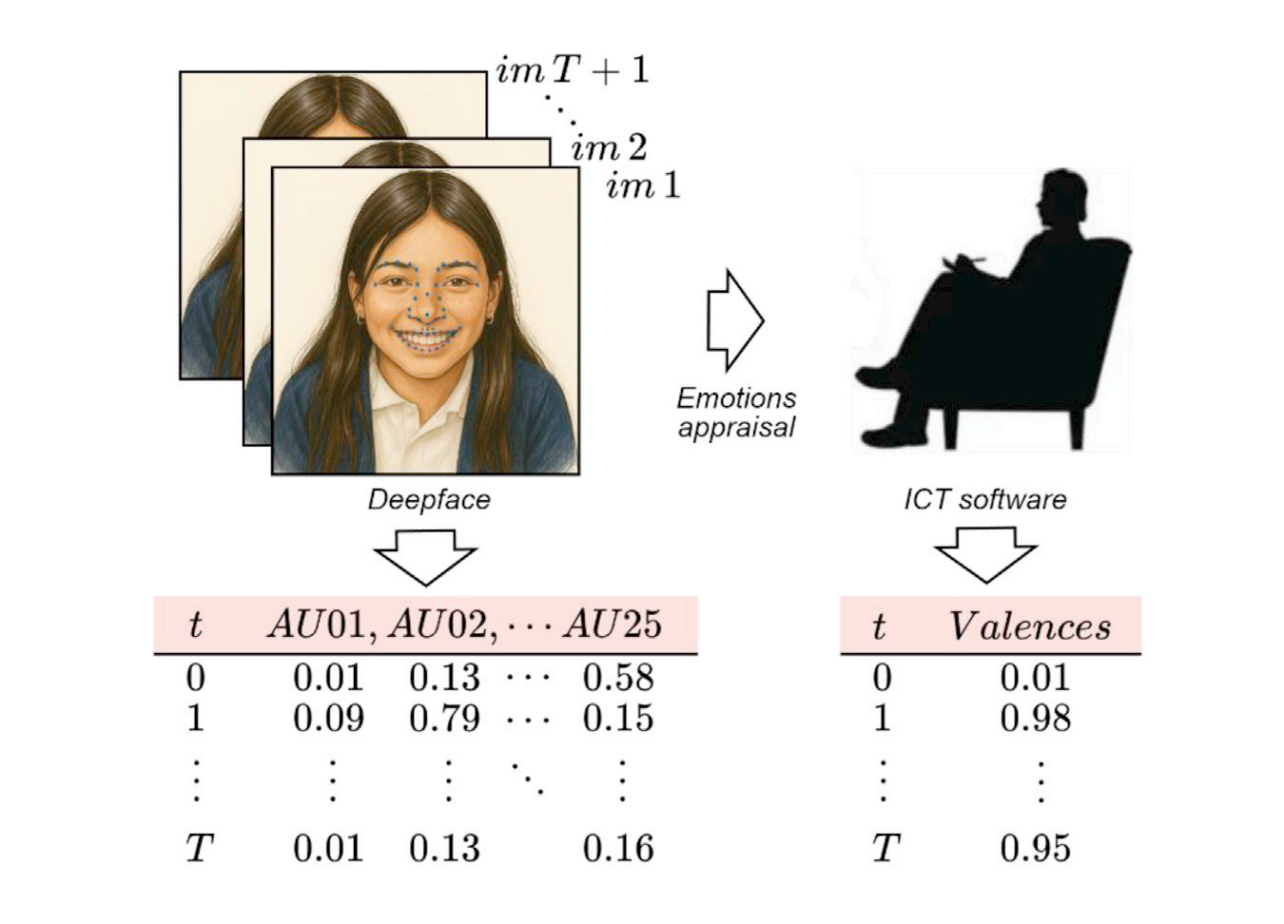
Correspondence between AUs and valence values.

Fig. 1. presents a diagram illustrating the process of emotion appraisal based on facial expressions. On the left, a sequence of video frames shows a girl with facial landmarks marked on her face, enabling the detection of AUs through *Deepface* Library. These landmarks are transformed into time-series feature vectors, generating files containing AU values per frame (AU01, AU02, …, AU25). Furthermore, the sequence of video frames is assessed by several experts, who assigns emotional valence scores over time. These scores indicate whether the expressed emotion is positive, negative, or neutral, and are stored in CSV files matched with the AU files. At the bottom, the timeline shows the correspondence between AU features and valence values at each time step.

It should be noted that although 22 students participated in the study for the collection of valence ratings, some videos were not considered due to a lack of consensus in the valence assessment, the child’s posture preventing reliable recognition of AUs, or technical issues during processing. For this reason, certain videos were not included in the analysis. Furthermore, to improve the interpretation of valence ratings according to the participants’ gender, the following information is provided:

• ID number of female participants: 01, 04, 05, 06, 08, 09, 10, 12, 13, 14, 16, 17.
• ID number Male participants: 02, 03, 07, 11, 15, 18, 19.

## 4 Experimental Design, Materials and Methods

**Fig. 2:**
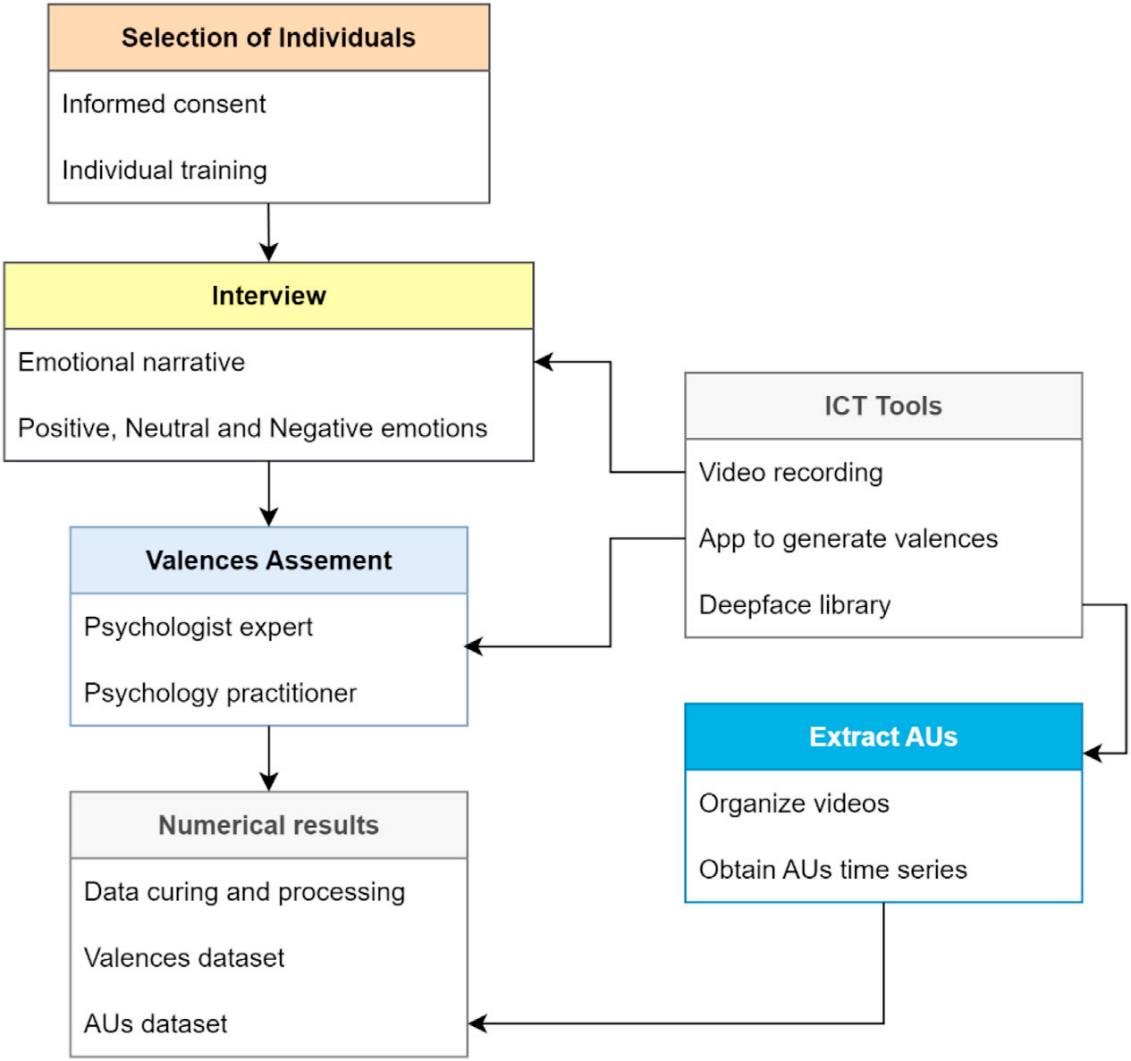
Methodology for evaluation of emotions perceived in children.

The following description outlines the methodology used for the evaluation of emotions perceived in children by means of emotional narrative. Initially, subjects were selected from an educational institution, to whom the purpose of the project was explained and who were asked to sign an informed consent form. Following the definition of the sample, interviews were conducted using the emotional narrative technique. In this technique, psychologists evoke three kind of emotions (negative, neutral and positive) in the participants. These interactions were meticulously recorded, thus forming a comprehensive data set. This data set was then subjected to rigorous evaluation by several experts, who assigned a series of valences to each video. In parallel, Facial Action Units [4] were extracted from each of the videos using the DeepFace library [3]. Finally, the raw valence and AU datasets were obtained. Each of these underwent a data review to equalize and synchronize the time series of AUs and Valences, erroneous series or those with incomplete data were removed, and error indices were calculated as a performance metric to indicate the success of the emotion evaluation. Fig. 2 presents the methodology step by step in detail.

### 4.1 Sampling and selection of individuals

A sample of sixth and seventh grade high school students from an Educative Institution located in Túquerres, Nariño, who voluntarily participated in the recorded interviews, was selected. The selection considered factors such as gender, age, and socioeconomic context to enrich the analysis. It should be mentioned that prior to their participation, the students' guardians signed an informed consent for the collection of personal information. In total, 22 students participated, 9 boys and 13 girls with ages from 10 to 12 years old. At this stage, children are capable of expressing basic emotions through facial cues; however, happiness is often expressed more vividly through bodily actions such as laughter, shouting, or clapping, due to their psychomotor development and limited internalization of social norms. While universal facial patterns of emotions like happiness can still be identified and coded, including body movements and verbal cues provides a more comprehensive understanding of emotional expression in this age group.

Additionally, research indicates that gender differences play an important role in emotional expression, as boys are generally less expressive, smile less, and exhibit less happiness than girls. Considering these differences, along with the tendency of children to express joy more through bodily actions, is essential for interpreting the results. Therefore, both gender-related factors and multimodal expressions must be taken into account in the subsequent analysis to ensure an accurate understanding of the emotional responses observed.

The environment was designed in a clean, standardized manner, with a black background that eliminated distractions, and the cameras were positioned facing forward to capture facial expressions up to the shoulders. This was done in order to minimize elements that could bias the training data and ensure that the model generalizes adequately to different populations.

### 4.2 Psychologist-guided interviewing using emotional narrative

To better understand human emotions in real life, a dataset was collected that captures natural and spontaneous emotional expressions. This work is based on the emotional narratives methodology developed (SENDv1) at Stanford University, more details in [2]. The SENDv1 dataset consists of videos of people, in this case children, sharing personal life stories that evoke various emotions. These spontaneous narratives not only capture natural emotional expressions, but also contain rich semantic and conceptual content. Moreover, these stories show a variety of emotional “trajectories” over time, providing a comprehensive and detailed dataset for modeling and time-series analysis of emotions.

Following the SENDv1 methodology, children were interviewed individually. During the interviews, they were asked to think of three very positive and three more negative events and were recorded while talking about them. The recording was self-paced: the experimenter let them talk as long as they wanted about each event, allowing them to capture natural and spontaneous emotional expressions of the participants.

The video interviews were guided by psychologists who used techniques to encourage positive or negative emotions in the participants. These techniques included: i) Emotional intelligence, which involves empathizing with the subject and recognizing their emotions to manage them effectively. ii) Active listening, which requires attentive listening and showing empathy to encourage a reaction from the interviewee. iii) Storytelling, which facilitates emotional connection and understanding between the interviewer and interviewee. iv) Reflective questions, which encourage emotional expression and self-awareness in the interviewee.

### 4.3 Evaluation of emotions by valence and indicators

**Fig. 3:**
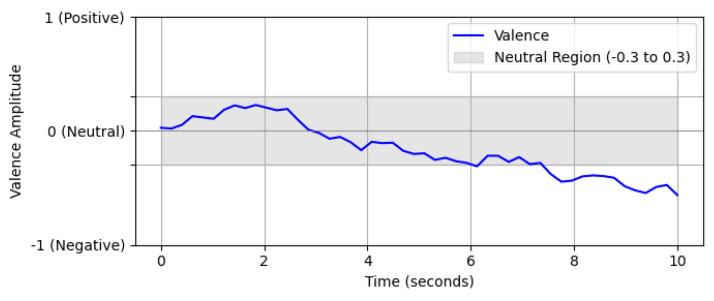
Valences curve.

The data were evaluated using valence scales to create a model that measures the identified emotions. A valence scale is a tool that is utilized for the purpose of measuring the positive or negative emotional charge exhibited in personal narratives. This measurement facilitated the capture of both the strength and the changes in emotions over time, thereby providing a more comprehensive understanding of the emotional experiences of the individuals sharing their personal narratives. After the compilation of the video set, a team of expert psychologists undertook a rigorous evaluation of the children's emotional responses as they share their narratives. This evaluation was conducted using a valence scale, a methodical tool designed to quantify the intensity and positivity of emotional responses. These ratings, ranging from "Negative" to "Positive", are recorded at one-second intervals, thereby creating continuous valence ratings for each video. Fig. 3 represents a time series of valences.

Subsequent to the establishment of the valence scale, trained raters evaluate each video, thereby producing a valence series that corresponds to the video's duration. In this study, $V_{N}^{P}\left(k\right)$ is used to denote the valence at each designated time point (k), P denotes the professional that evaluated the video, and N represents the student who corresponds the valence curve. The average weighted valence for an individual can then be defined as:

$${\bar{V}}_{n}\left(k\right)=\alpha_{p}\sum_{p=1}^{P}V_{n}^{p}\left(k\right)$$

The weighting for professional p, designated ($\alpha_{p}$), is determined by their expertise in recognizing emotions in facial expressions. The average weighted valence has been shown to facilitate the generalisation of an individual's series of emotions, thereby creating a curve that demonstrates the dynamics and changes in emotions that professionals observe. These ratings facilitate the creation of machine learning models that can more accurately predict and comprehend human emotions in various scenarios.

It is important to note that the results were averaged and adjusted to incorporate all the evaluations provided by the experts. This means that the Valence files contain the average valence scores obtained. This process resulted in a more valid and reliable measure, thereby supporting more accurate and relevant learning by the emotion detection algorithm.

Conversely, consensus error ($e_{c}$) is defined as a metric that quantifies the dispersion or alignment of the observers' valences from the mean valence. The consensus error is defined as follows:

$$e_{c}=\frac{1}{\mathrm{PK}}\sum_{p=1}^{P}\sum_{k=1}^{K}\left|{\bar{V}}_{n}\left(k\right)-V_{n}^{p}\left(k\right)\right|$$

Consensus error reflects the reliability of video interpretation. Low error means observers agree on the expressed emotions, indicating confidence in the assessment. High error suggests disagreement, possibly because the emotion wasn't clear. In such cases, removing the video is recommended to ensure accurate emotional ratings. Consensus error is a measure that ensures the dataset's quality and reliability. It helps identify and remove videos where expert observers had uncleared or inconsistent emotional perceptions. This ensures the final dataset is high quality and suitable for training machine learning models.

### 4.4 AUs extraction

The extraction of the Facial Action Units was facilitated by the utilization of the DeepFace library, a sophisticated software that has been demonstrated to achieve a high degree of precision in the identification of over 15 AUs, with a success rate exceeding 97 % [3]. The library was utilized to review each of the images in the videos, with the objective of identifying the series of AUs that exhibited a congruent sampling rate with that of the valences evaluated in the videos. In this particular case, twelve AU values were extracted per frame (AU01_r, AU02_r, AU04_r, AU05_r, AU06_r, AU07_r, AU12_r, AU14_r, AU15_r, AU17_r, AU20_r, AU25_r). The AUs employed in this particular dataset correspond to specific facial muscle movements that have been encoded by the FACS system. AU01_r is the designation for the Inner Brow Raiser, while AU02_r is the designation for the Outer Brow Raiser. AU04_r is indicative of the Brow Lowerer (associated with frowning). AU05_r is the Upper Lid Raiser, and AU06_r represents the Cheek Raiser (engaging the orbicularis oculi muscle). AU07_R is the designated reference for the Lid Tightener. On the lower face, AU12_r corresponds to the Lip Corner Puller (a common feature in smiling) and AU14_r is the Dimpler (often associated with contempt). AU15_r denotes the Lip Corner Depressor, while AU17_r signifies the Chin Raiser. AU20_r corresponds to the Lip Stretcher, and finally, AU25_r represents the Lips Part (mouth opening). The selection of these AUs was made on the basis that they facilitate the articulation of a broad spectrum of emotions, encompassing both positive and negative sentiments.

### 4.5 ITC tools

Video recording: The interviews were done in an office that was 3.5 m x 3.2 m and had good lighting. A 5-megapixel SV3C IP camera (model B06W-5MP-HX) was used to send video using RTSP over Ethernet or WiFi. The camera was placed at the top of the office and recorded the students' faces in videos lasting between 60 and 180 s. The camera had a resolution of 1920×1088 at 30 frames per second.

Software Development Technologies: HTML5 and CSS3, these are used to design the user interface, making it look good and working well so that users can interact with it. JavaScript: It can be used for interactive slider functions, video loading and data handling, and allows real-time user input processing. AJAX lets you update web pages right away. It does this by sending information to the web application. This makes the web application easier to use and faster. Bootstrap: A web design framework that makes it easy to create a responsive website with pre-designed tools and components, providing an efficient user interface for evaluators.

How to use it: The main screen of the software has a video section and a slider. The video shows the interview, while the slider lets people indicate how they feel. It is very important that the user interface is easy to use so that people can interact with it intuitively and efficiently. Design principles like Jakob Nielsen heuristics and user-centred design help to make sure that users have a good experience. The slider lets people rate emotions from bad to good. Slider data is stored in a database so that it can be analyzed. This emotion valence system is based on Russell's affective space theory, which represents emotions in a two-dimensional space of strength and energy for accurate categorization. Emotions were shown on a scale from −1 to 1, with −1 being the most negative and 1 being the most positive.

## Limitations

It is important to note that the generalizability of the findings is potentially limited due to the inclusion of only 22 children in the dataset, because of ethical and logistical constraints. Despite the dataset comprising 100 time series with a duration of 10 s each, on average, the number of unique participants is relatively small. However, this issue can be addressed through the application of data augmentation techniques.

The data was collected in a controlled environment within a single geographic region, which could introduce cultural or contextual bias. These limitations must be taken into account when reusing the dataset for training emotion recognition models or conducting comparative studies.

Finally, one limitation of this study is that the dataset has not been externally validated; valence ratings were derived from expert observations rather than real-life experimental evaluations.

## Ethics Statement

This study involved children between 10 and 12 years of age. Written informed consent was obtained from the legal guardians of all participants prior to data collection. The dataset consists solely of anonymized time-series data derived from facial Action Units and expert-rated valence scores. No images, audio, names, or personally identifiable information are included. Formal ethical committee approval was not required under local institutional regulations. All procedures were conducted in accordance with the principles outlined in the Declaration of Helsinki.

## CRediT Author Statement

**John Barco-Jiménez:** Conceptualization, Methodology, Data curation, Writing. **Sixto Campaña:** Conceptualization, Methodology. **Álvaro Cervelión:** Data curation, Investigation: **Harold Cabrera:** Software, Visualization, Investigation. **Carlos Tobar:** Conceptualization, Methodology, Validation, Writing. **Roberto Jaramillo:** Conceptualization, Methodology, Validation, Writing. **Andrés Díaz:** Conceptualization and Methodology. **Abel Méndez Porras:** Writing- Reviewing.

## Data Availability

Mendeley DataChildren’s Emotional Expression Dataset: Action Units and Valence Time Series (Original data)
